# Thiols Act as Methyl Traps in the Biocatalytic Demethylation of Guaiacol Derivatives

**DOI:** 10.1002/anie.202104278

**Published:** 2021-06-29

**Authors:** Simona Pompei, Christopher Grimm, Christine Schiller, Lukas Schober, Wolfgang Kroutil

**Affiliations:** ^1^ Institute of Chemistry, Biocatalytic Synthesis University of Graz, NAWI Graz Heinrichstrasse 28 8010 Graz Austria; ^2^ BioTechMed Graz 8010 Graz Austria; ^3^ Field of Excellence BioHealth-University of Graz 8010 Graz Austria

**Keywords:** biotransformation, cobalamin-dependent enzymes, demethylation, methyl phenyl ethers, thioethers

## Abstract

Demethylating methyl phenyl ethers is challenging, especially when the products are catechol derivatives prone to follow‐up reactions. For biocatalytic demethylation, monooxygenases have previously been described requiring molecular oxygen which may cause oxidative side reactions. Here we show that such compounds can be demethylated anaerobically by using cobalamin‐dependent methyltransferases exploiting thiols like ethyl 3‐mercaptopropionate as a methyl trap. Using just two equivalents of this reagent, a broad spectrum of substituted guaiacol derivatives were demethylated, with conversions mostly above 90 %. This strategy was used to prepare the highly valuable antioxidant hydroxytyrosol on a one‐gram scale in 97 % isolated yield.

## Graphical abstract

Efficient demethylation of methyl phenyl ethers was achieved by biocatalytic O_2_‐free methyl transfer to thiols forming thio ethers. Since the methyl group is not cleaved from the sulfur under these conditions, the thiol compound acts like a trap driving the reaction towards completion.

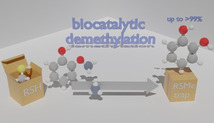

The phenolic functionality is present in many pharmacophores of both natural and synthetic origin.^[1]^ Consequently, phenolics are of interest for pharma, human nutrition and toxicology.^[2]^ Many biological activities are attributed to phenols like anti‐inflammatory, antimicrobial, antiviral and antitumor properties among others.^[1a]^ Moreover, 1,2‐diphenols—catechols—play an important role in the synthesis of fine‐chemicals, adhesives, coatings, rubber and plastic products, as well as in photography.^[3]^ The chemical synthesis of many of these compounds often requires protecting groups to tame the reactivity during other transformations. The most common masking strategy for this group is the etherification.

The ether functionality, especially methyl ethers, is rather inert under various conditions and therefore protects the otherwise easily oxidable catechol moiety; yet, this inertness leaves the ether functionality difficult to remove, unless harsh conditions are applied (acid or base).^[4]^ Since the methyl ether group is widely found in nature,^[5]^ a variety of enzymes are able to transform this moiety such as (i) monooxygenases,^[6]^ (ii) peroxygenases,^[7]^ (iii) dehydratases as observed for PEG degradation^[8]^ and (iv) methyltransferases.^[9]^ Mostly, the methyl ether groups are cleaved by P450 enzymes at the expense of NAD(P)H and molecular oxygen by C−H oxidation at the carbon next to the ether oxygen, resulting in a hemiacetal, which then decomposes.[^6a^, ^7c^, ^10^] However, the oxidative conditions may cause various challenges;[^7c^, ^11^] e.g., when catechol is the target product, the presence of molecular oxygen may initiate undesired follow‐up reactions (such as polymerization, autooxidation, quinone formation). On the other hand, homoacetogenic bacteria are capable of growing on methyl‐aryl ethers,^[5]^ degrading these compounds as a source of energy. These bacteria use methyltransferases to shuttle the methyl group to an acceptor molecule (e.g. tetrahydrofolate‐THF)^[12]^ via methylcobalamin bound to a carrier protein (CP).^[13]^

Previously, we showed that these cobalamin methyltransferases (cob‐MT) are able to shuttle the methyl group between structurally related molecules, thus from guaiacol derivatives to catechol derivatives. However, that reaction was limited by its equilibrium (Scheme 1 A);^[14]^ By omitting a methyl acceptor, isomerization, and thus intramolecular methyl transfer, was observed (Scheme 1 B).^[15]^ This isomerization was also a prominent side reaction in the case of the intermolecular methyl transfer due to equilibria. Furthermore, in the intermolecular methyl transfer, the structural similarity between donor and acceptor led to a mixture of products, which was difficult to separate, resulting in poorer yields.

**Scheme 1:**
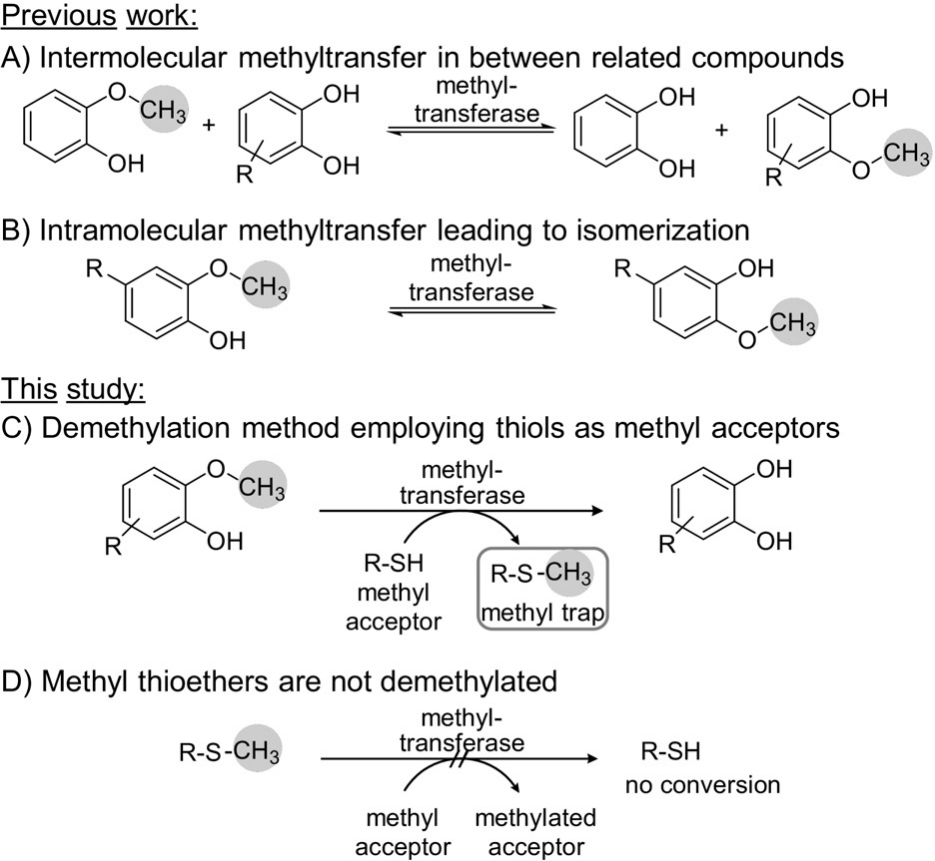
Demethylation/Isomerization employing cobalamin methyltransferases; A) equilibrium in intermolecular demethylation of 2‐methoxyphenol employing catechols as methyl acceptors; B) intramolecular isomerization; C) quasi‐irreversible demethylation of substituted guaiacol derivatives employing thiols as methyl acceptors; D) methyl thiols are not demethylated acting therefore as quasi‐irreversible methyl traps.

Here, we report on the identification of methyl acceptors acting as methyl traps (Scheme 1 C). In other words, the methyl moiety is quasi irreversibly bound to the acceptor (Scheme 1 D), thereby shifting the equilibrium of the demethylation, and reducing the amount of reagent needed.

As thiols are used in nature as methyl acceptors for detoxifying hydrogen sulfide or xenobiotic thiols^[16]^ during methanogenesis (e.g. coenzyme M)^[17]^ or in methionine synthesis,^[18]^ we wondered whether thiols may serve as suitable methyl acceptors for cobalamin‐dependent demethylation by methyl transferases. Consequently, various thiols were investigated as potential methyl acceptors for demethylating guaiacol **1 a** as test substrate, using the cobalamin‐dependent methyltransferase I from *Desulfitobacterium hafniense* (*dhaf*‐MT)^[14b]^ as cell‐free extract (Scheme 2, for detailed methods see Supporting Information). We investigated a library of thiol compounds encompassing carboxylic acids (**3 a**, **3 f**), esters (**3 b**–**c**,**e**), aromatic thiols (**3 d**,**j**) and di‐thio compounds (**3 g**–**j**, Table S1).

**Scheme 2:**
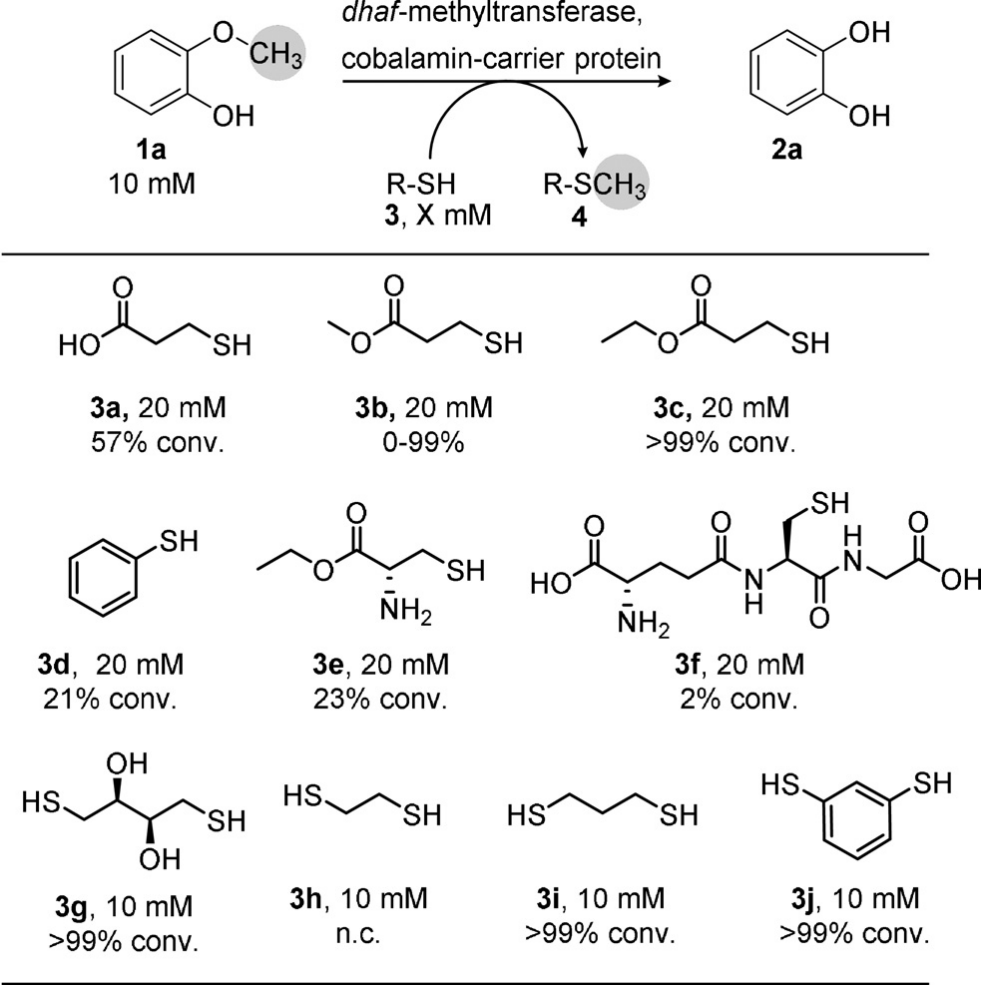
Oxygen‐free biocatalytic demethylation of guaiacol **1 a** using various thiols as methyl acceptor. Reaction conditions: MOPS buffer (50 mM, 150 mM KCl pH 6.5), MTase I (50 mg mL^−1^ CFE ≡ 1.95 mg mL^−1^ MTase I) and CP (500 μL mL^−1^ reconstituted *holo*‐CP solution ≡ 21 mg mL^−1^ CP), 800 rpm, 30 °C, 24 h. For **3 h**–**3 j** 10 % v/v DMSO was present in the reaction mixture (DMSO was needed for pre‐dissolving the di‐thiols).

To our delight, it turned out that the methyl transferase *dhaf*‐MT is not limited to catechols as acceptors, as previously reported,[^14^, ^15^] but also accepts thiol compounds. Using two equivalents of 3‐mercaptopropionic acid **3 a** resulted in 57 % conversion. Taking the corresponding methyl ester **3 b** led to varied results when using the biocatalyst as cell‐free extract, due to concomitant hydrolysis of the methyl ester (Figure S21). Seeking an ester less prone to hydrolysis, the corresponding ethyl ester **3 c** was investigated which led to quantitative conversion of substrate **1 a** with only two equivalents of **3 c**. The corresponding methylated thio‐ether **4 c** was separately tested to examine whether it is demethylated when using catechol **2 a** as acceptor; interestingly, no demethylation was found, indicating that, under the conditions employed, **3 c** may act as a quasi‐irreversible trap for the methyl group (Scheme 1 D, Figure S20). Thiols **3 d**–**f** were clearly inferior as methyl acceptors. Dithiols **3 g**–**j** were tested at a 1:1 ratio with the substrate to have the same concentration of thiol groups as in the previous experiments. While **3 h** did not react at all, the other di‐thiols **3 g**,**i**,**j** allowed to run the demethylation reaction to completion. Although these di‐thiols seemed to react efficiently, DMSO was needed as a co‐solvent in the reaction due to insolubility of the acceptors **3 i** and **3 j**; moreover, **3 g** and its corresponding methylated derivatives led to analytical challenges. For these reasons, ethyl 3‐mercaptopropionate **3 c** was used for further experiments.

To learn about the influence of the amount of methyl acceptor on the outcome of the reaction and whether substituted guaiacol derivatives are also transformed under these conditions, the demethylation of homovanillyl alcohol *m*‐**1 b** (10 mM) was investigated at varied equivalents of methyl acceptor **3 c**. Above two equivalents of **3 c**, the reaction went in general to completion within 24 hours (Figure 1), while at two equivalents the reaction reached almost completion (98 % conv.). On the other hand, at 1.5 equivalents of **3 c** the reaction mixture contained 67 % of demethylated product **2 b** as well as 18 % of the isomerized substrate *p*‐**1 b** and 15 % remaining substrate *m*‐**1 b**. Consequently, two equivalents of methyl acceptor seemed to be a good compromise to achieve high conversion with a minimum amount of thiol within 24 hours. Nevertheless, depending on the requirements of a reaction, just using *one* equivalent of a di‐thio compound might be desired for certain applications.

**Figure 1:**
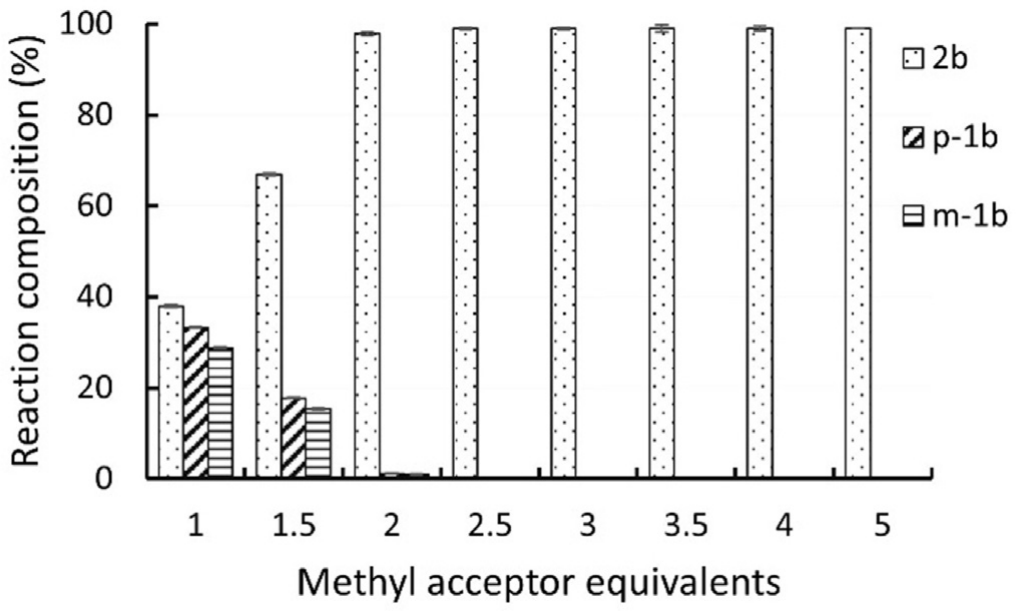
Demethylation of homovanillyl alcohol *m*‐**1 b** (10 mM) to **2 b** at varied equivalent of thiol **3 c** as methyl acceptor after 24 h. Reaction conditions: MOPS buffer (50 mM, 150 mM KCl, pH 6.5), *dhaf*‐MT (40 mg mL^−1^ CFE ≡ 1.56 mg mL^−1^ *dhaf*‐MT) and CP (400 μL mL^−1^ reconstituted *holo*‐CP solution ≡ 21 mg mL^−1^ CP), 30 °C, 800 rpm. Experiments were performed in triplicate.

We then investigated a broad range of substituted guaiacol derivatives possessing the substituent either *para*‐ or *meta*‐ to the methoxy group, using two equivalents of methyl acceptor **3 c**, (Scheme 3). In most cases conversions above 90 % were reached (see Supporting Information, Table S4‐S5). Only for the carbaldehydes (*m*‐ and *p*‐**1 e**) a lower conversion was achieved (74 and 64 %, respectively). Furthermore, the unwanted isomerisation product was below 10 % or not detectable at all for all substrates. Note that the isomerisation product, as can be seen from the substrates (compare *m*‐ versus *p*‐**1**), is also demethylated; thus, the isomerization is a reversible side reaction, finally allowing the demethylation to run to completion. Moreover, besides the *meta*/*para* substituted derivatives, an *ortho* substituted guaiacol, namely 2‐methoxy‐3‐methylphenol was also investigated. In this case, quantitative conversion (>99 %) was observed, indicating an even broader substrate scope.

**Scheme 3:**
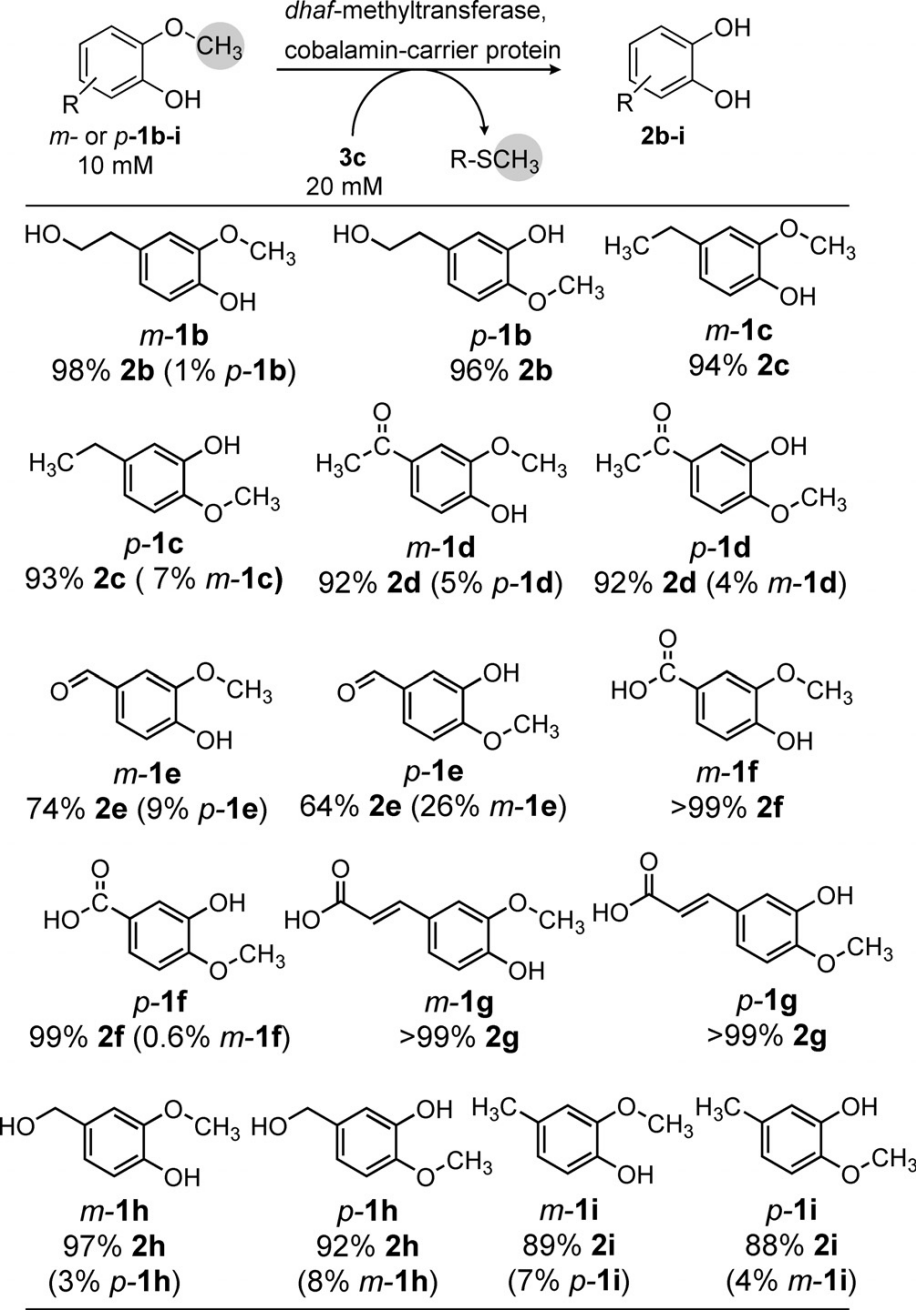
Biocatalytic demethylation of *m*‐ or *p*‐substituted guaiacols **1 b**–**i** employing thiol **3 c** as methyl sink. Besides the demethylation product **2**, isomerization of the substrate was observed in some cases by moving the methyl group to the neighbouring phenol group. Percentage of product **2** is reported below each substrate number; the amount of isomerization product in the reaction mixture is in brackets. Reaction conditions: MOPS buffer (50 mM, 150 mM KCl pH 6.5), *dhaf*‐MT (50 mg mL^−1^ CFE ≡ 1.95 mg mL^−1^ *dhaf*‐MTase I) and CP (500 μL mL^−1^ reconstituted *holo*‐CP solution ≡ 21 mg mL^−1^ CP), 800 rpm, 30 °C, 24 h.

The product of the demethylation of *m*‐ or *p*‐**1 b** is hydroxytyrosol **2 b**, which is found in nature in olive leaves, fruits, and extra virgin olive oil. This natural product is well‐known as one of the most powerful antioxidants found in nature,^[19]^ conferring on cells protection from free radicals.^[20]^ Additionally, several other biological activities have been uncovered through the years.^[20c]^ Due to the extraordinary properties of this compound, numerous efforts have been made for its production using chemical as well as biotechnological approaches.

While the majority of natural hydroxytyrosol **2 b** is derived from olive oil,^[21]^ the chemical synthesis of **2 b** has been tackled by many researchers over the last decades. From its first synthesis in 1949,^[22]^ where hydroxytyrosol was produced by reducing 3,4‐dihydroxyphenylacetic acid using LiAlH_4_, many more synthetic strategies have been established,^[23]^ some of which use greener methods.^[24]^ Nevertheless, most synthetic efforts are still limited by either low yield and/or multistep syntheses.

The biotechnological production of hydroxytyrosol has also been reported. Most strategies involve tyrosinases, exploiting whole‐cell machineries as well as cell‐free biocatalysts.[^23b^, ^25^]

Since hydroxytyrosol **2 b** is currently priced at about 200 times that of *m*‐**1 b**,^[26]^ the anaerobic demethylation reaction of *m*‐**1 b** leading to **2 b** was tested for the possibility to perform it on a gram scale. Firstly, we prepared the catalyst and performed the reaction in a similar fashion as on analytical scale, but with an increased amount of substrate (40 mg *m*‐**1 b**) and catalyst. Results showed that this approach was feasible (see “Semi‐preparative scale biotransformation, 24 mL” in Supporting Information for details). However, for larger scale, the preparation of the catalyst is rather tedious due to the loading procedure required for the carrier protein with cobalamin, as this usually involves a desalting step. To simplify the procedure, the desalting step was omitted, and the same good results were obtained (Table S6). After further optimizing the experimental procedure on a 0.25 g scale (see “Biotransformation semi‐preparative, 150 mL” in Supporting Information for more details), the demethylation was finally shown for one gram of *m*‐**1 b**. After 25 hours, HPLC analysis indicated quantitative conversion. Extraction and purification via column chromatography afforded pure **2 b** in 97 % yield (886.5 mg, productivity 1.44 g L^−1^/d, see Supporting information). In comparison to the biocatalysis literature, where the hydroxylation of tyrosol has largely been reported,^[23b]^ this represents a unprecedent high yielding approach. This result can be attributed to the mild conditions in the oxygen‐free, one step demethylation procedure using the mercapto ester **3 c** as methyl trap.

In summary, an efficient biocatalytic oxygen‐free method for demethylating methyl phenyl ethers, exemplified for guaiacol derivatives, is reported here using thio compounds, preferentially ethyl 3‐mercaptopropanoate **3 c**, as methyl trap. The one pot protocol was shown to be applicable for a broad scope of substituted guaiacol derivatives, whereby many of them were transformed with a conversion exceeding 90 %, at 30 °C and mild pH (pH 6.5) in buffer. Furthermore, the approach should be extendable to other cobalamin dependent methyltransferases possessing different preference for the substrate pattern.^[27]^ We envisage that the substrate scope could be broadened by enzyme engineering. Having improved the procedure also for preparative scale, the highly valuable antioxidant hydroxytyrosol **2 b** was prepared on a one‐gram scale with 97 % isolated yield. The study shows that biocatalytic demethylation under anaerobic and mild conditions of methyl phenyl ethers has now become an alternative method to be added to the toolbox of organic chemistry.

## Conflict of interest

The authors declare no conflict of interest.

## Supporting information

As a service to our authors and readers, this journal provides supporting information supplied by the authors. Such materials are peer reviewed and may be re‐organized for online delivery, but are not copy‐edited or typeset. Technical support issues arising from supporting information (other than missing files) should be addressed to the authors.

Supplementary
