# Supplementary material for: Thiols Act as Methyl Traps in the Biocatalytic Demethylation of Guaiacol Derivatives
Source: Angew Chem Int Ed Engl. 2021 Jun 29;60(31):16906–10. doi: 10.1002/anie.202104278 (PMC8361964; doi:10.1002/anie.202104278)
Supplement: Supplementary file 1 — Supplementary [file ANIE-60-16906-s001.pdf]

Angewandte Chemie
Eine Zeitschrift der Gesellschaft Deutscher Chemiker

Supporting Information

Thiols Act as Methyl Traps in the Biocatalytic Demethylation of Guaiacol Derivatives

*Simona Pompei, Christopher Grimm, Christine Schiller, Lukas Schober, and Wolfgang Kroutil**

anie_202104278_sm_miscellaneous_information.pdf

Table of Contents

Experimental Procedures 3
Supplementary Results 6
Calibrations Curves and Chromatograms 8
NMR 22
References 23

Experimental Procedures

General Information

^{1}H- and ^{13}C-NMR spectra were recorded at 20°C on a 300 MHz Bruker NMR. The conversion of all biotransformations was measured by reversed phase HPLC with an Agilent 1260 Infinity HPLC system at 25°C, using a Phenomenex Luna® 5 mm, C18, 100 A (250 × 4.6 mm) column. Detection was performed with a diode array detector (G4212B) and quantification was achieved by calibration with standards. Alternatively, an Agilent 7890 A GC system together with a mass-selective Agilent 5975 detector (electron impact ionization, 70 eV, mass scan= 33-400 m/z) was used for detection. For separation, an Agilent J&W HP-5ms column (30 m, 250 µm, 0.25 µm) and helium (0.7 mL/min) as carrier gas were employed. TLC was carried out with pre-coated aluminum sheets (TLC Silica gel 60 F254, Merck) with detection by UV (254 nm) and/or by staining with cerium molybdate solution. All chemicals and solvents were obtained from commercial suppliers (TCI, Sigma Aldrich/Fluka, VWR International/Merck, Roth) and used as received unless stated otherwise.
Enzymes were recombinantly expressed in *E. coli* and used as lyophilized cell-free extract (CFE) or purified preparation. The corrinoid protein (CP) was reconstituted with methylcobalamin prior to use to yield active *holo*-CP. Biocatalytic reactions were performed in triplicates in degassed buffers under inert atmosphere, using a Labstar MB10-compact glovebox (MBraun, Germany) equipped with a special O_2-sensor (MB-OX-EC) to be used with aqueous systems.

Enzyme Expression

Expression of required proteins was performed with *E. coli* using the plasmids pEG457 and pEG458 (dhaf4610 MTase I and dhaf4610-strep tag MTaseI) and pEG459 and pEG460 (dhaf4611 CP and dhaf4611-strep tag CP) as previously reported.[1] Over-night cultures (ONCs) were prepared in 50 mL falcon tubes by supplementing LB-medium (20 mL) with ampicillin (100 µg/mL). After inoculation with a single colony of transformed cells the cultures were incubated overnight at 37 °C and 120 rpm. Main cultures (500 mL volume in 2 L non-baffled Erlenmeyer flask) supplemented with ampicillin (100 µg/mL) were inoculated with the appropriate volume of ONC to an initial OD_{600} of 0.05. After incubation at 37 °C and 120 rpm (2 hours) until an OD_{600} of 0.6-0.8 was reached, expression of the target protein was induced by the addition of AHTC (0.2 µg/mL). Shaking was continued at 120 rpm and 25 °C overnight. After shaking for 24 h, the cells were harvested by centrifugation (4,000 rpm, 4 °C, 20 min). The cell pellets were re-suspended in 50 mM TRIS-HCl buffer pH 7 yielding a 15 wt % cell suspension. Next, the cells were disrupted by sonication (Branson Digital Sonifier®) using the following settings: duration 2 min, 2.0 s sonicate, 1.0 s pause, 40% amplitude. While sonication was performed (at least 3 times with 30 seconds breaks in between), the cells were constantly cooled with ice. Afterwards by centrifugation for 15 min at 14,500 rpm, the cell fragments were removed from the extract. The cell-free extract was finally lyophilized and stored at 4 °C, ready to be used for biotransformations or further used for the purification step. The protein content of the single fraction of MTI (Figure S1) amounted to 0.81 µg which was calculated by densitometry (ImageJ).

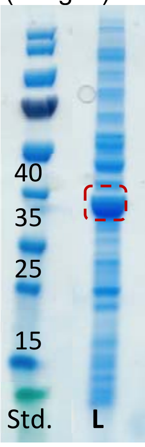

Figure S1. SDS-PAGE analysis of the expression of MTase I in *E. coli* BL21 (DE3) pLysS for its evaluation with ImageJ. After cell disruption via sonication, the soluble lysate was analyzed and it is reported as (L). Std.: PageRulerTM Prestained Protein Ladder.

Protein Purification

The strep-tag purification of dhaf4610 and dhaf4611 was performed with Strep-Tactin® XT High-Capacity Purification kit according to the manual provided by the supplier. After the strep-tag column

had been equilibrated with 2 column bed volumes (CVs) of Buffer W (100 mM TRIS/HCl, pH 8, 150 mM NaCl), the cell-free extract was applied to the column and the flow-through (FT) was collected. Then unbound protein was removed by washing the column with 5 CVs of Buffer W and the wash fraction (W) was again collected. The bound protein was eluted by adding 6x 0.5 CVs of Buffer BTX (100 mM TRIS/HCl, pH 8, 150 mM NaCl, 2.5 mM biotin) to the column and collected in 2.5 mL fractions. The protein content of the fractions was determined by means of a photometric Bradford assay. The purified protein solution of dhaf4610 and/or dhaf4611 was rebuffered into MOPS/KOH buffer (50 mM, pH 7, 150 mM KCl) using a PD10 column and concentrated to about 2.5 mL using Vivaspin vials. Afterwards, the purified protein was finally lyophilized and stored at 4°C until further use.

HPLC analysis
After the biotransformation (usually in 120 µL total volume, unless stated otherwise), an aliquot of 90 µL from the sample was taken and the reaction was quenched by the addition of acetonitrile (MeCN, 540 µL). After incubation (30 min) at room temperature, HPLC grade H_2O (270 µL) was added, and denatured protein was removed by centrifugation (14,000 rpm, 15 min). The supernatant was filtrated through a pipette tip filled with cotton and then the HPLC analysis was performed. A reverse phase method was used employing LUNA C18 as a stationary phase and water and MeCN both containing 0.1% trifluoroacetic acid (TFA) as mobile phase with a flow rate of 1 mL/min. In the standard method (method A) 100% H_2O were held for 2 min, then a gradient from 0% MeCN to 40% MeCN over 13 min was applied, followed by a gradient to 100% MeCN within 5 min, which was held for 2 min. Finally, 100% H_2O were held for 3 min. Another method used (method C) was as follows: the column was rinsed with 100% H_2O for 2 min, then a gradient from 0% MeCN to 30% MeCN over 13 min was applied, followed by a gradient to 100% MeCN within 5 min, which was held for 2 min. Lastly 100% H_2O were detained for 3 min. Other two methods used for the new substrates screened are method D (0-70 gradient of MeCN, 25 min in total).

GC-MS analysis
An Agilent 7890 A GC system together with a mass-selective Agilent 5975 detector (electron impact ionization, 70 eV, mass scan= 33-400 m/z) was used. For separation, an Agilent J&W HP-5ms column (30 m, 250 µm, 0.25 µm) and helium (0.7 mL/min) as carrier gas were employed. Samples (120 µL) from the biotransformation were extracted with EtOAc (2 x 120 µL), dried over Na_2SO_4, and injected into GC-MS (injection temperature 250 °C, split ratio 90:1).

Biotransformation

Preparation of *holo*-CP. The functional *holo*-CP was obtained by reconstitution of the corrinoid protein with exogenous cofactor (methyl cobalamin) under inert atmosphere. For this purpose, methyl cobalamin (2 mM) was dissolved in the presence of betaine (3 M) and DTT (2 mM) in TRIS/HCl buffer (50 mM, pH 7, 0.5 mM DTT, 0.1 mM PMSF) to form a reconstitution buffer. Then, freeze-dried CP (100 mg CFE) was loaded with the freshly prepared reconstitution buffer (1 mL) and incubated for at least 2 h at 4 °C to allow incorporation of the cofactor. Afterwards salts and unbound cobalamin were removed using a PD MidiTrap™ G-25 column (GE Healthcare) according to the manual provided by the manufacturer. Finally, reconstituted *holo*-CP was eluted with MOPS/KOH buffer (100 mM, pH 6.5, 150 mM KCl) yielding a red colored protein solution which was stored at 4 °C until further use. The content of *holo*-CP in CFE (21 mg/mL) was taken from literature.[2]

Biotransformation transmethylation analytical scale. Biocatalytic reactions were performed in degassed buffers under inert atmosphere (N_2 5.0) in a MBraun LABstar glove box equipped with a MB-OX-EC O_2-sensor. Analytical biotransformations were carried out in 1 mL Eppendorf Tubes® in 120 µL scale. The lyophilized MT I (crude extract 50 mg/mL ≡ 1.95 mg/mL *dhaf*-MTase I; 13 mg/mL for the experiment with purified enzyme) was rehydrated in *holo*-CP solution (500 µL/mL reconstituted *holo*-CP solution ≡ 21 mg/mL CP) and donor (10 mM) and acceptor (variable concentration, ranging from 10 to 50 mM) dissolved in MOPS/KOH buffer (50 mM, pH 6.5, containing 150 mM KCl) were finally added. The reaction mixture was then shaken in the glovebox at 30 °C and 800 rpm for 24 h. Afterwards, an aliquot of 90 µL from each sample was taken out and the reaction was quenched by adding acetonitrile (MeCN, 540 µL). After incubation (30 min) at room temperature HPLC grade H_2O (270 µL) was added, and denatured protein was removed by centrifugation (14,000 rpm, 15 min). The supernatant was filtrated through a pipette tip filled with cotton and then the HPLC analysis was performed.

Semi-preparative scale biotransformation (24 mL). Semi-preparative scale biotransformation (also prepared under inert atmosphere (N_2 5.0) in a MBraun LABstar glove box) was carried out on a 24 mL scale (using a 50 mL volume Schlenk Flask) as follows: freeze-dried MTase **I** (1.2 g, crude extract 50 mg/mL, 1.95 mg/mL *dhaf*-MTase I) was rehydrated in *holo*-CP solution (12 mL, 500 µL/mL reconstituted *holo*-CP solution ≡ 21 mg/mL CP, obtained with the reconstitution-desalting step protocol). Donor *m*-**1b** (40.3 mg, 10 mM) and acceptor **3c** (76 µL, 25 mM) dissolved in MOPS/KOH buffer (50 mM, pH 6.5, containing 150 mM KCl) were finally added accordingly. The reaction vessel was then sealed with a septum, covered with aluminum foil, and left shaking in an incubator at 30 °C and 120 rpm. After 24 h quantitative conversion was observed by HPLC-UV. The crude mixture was then distributed in four Falcon Tubes (6 mL in each 50 mL Falcon Tube) and extracted with EtOAc (5 mL x3) and centrifuged (4,000 rpm, 20 minutes, room temperature). The organic phases were combined and dried over Na_2SO_4. The solvent was finally evaporated under reduced pressure. After purification via column chromatography (1:1 EtOAc/cyclohexane), the isolated product **2b** was obtained in 56% yield as yellowish oil (20.8 mg). ^{1}H NMR (300 MHz, MeOD) δ 6.66 (m, 2H), 6.52 (m, 1H), 3.67 (t, J = 7.3 Hz, 2H), 2.66 (t, J = 7.2 Hz, 2H). ^{13}C NMR (75 MHz, MeOD) δ 146.1, 144.6, 131.7, 121.2, 117.0, 116.3, 64.6, 39.6.

Biotransformation semi-preparative (150 mL). Semi-preparative scale biotransformation (also prepared under inert atmosphere (N_2 5.0 bars, in a MBraun LABstar glove box) was carried out on 150 mL scale (using a 500 mL volume cultivating Flask) as follows: freeze-dried MTase **I** (7.5 g, crude extract 50 mg/mL ≡ 1.95 mg/mL *dhaf*-MTase **I**) was rehydrated in *holo*-CP solution (75 mL, 500 µL/mL reconstituted *holo*-CP solution ≡ 21 mg/mL CP, obtained omitting the desalting step protocol). Donor *m*-**1b** (252 mg, 10 mM) and acceptor **3c** (494 µL, 25 mM) dissolved in MOPS/KOH buffer (50 mM, pH 6.5, containing 150 mM KCl) were finally added accordingly. The reaction vessel was then sealed with a septum, covered with aluminum foil, and left shaking in an incubator at 30 °C and 120 rpm. After 24 h, the quantitative conversion was observed by means of HPLC-UV. The crude mixture was then divided in centrifuge vessels (15 mL per Falcon Tube). The aqueous mixture was then extracted with EtOAc (15 mL x3) while centrifuging (20 min, 4,000 rpm x3). The organic phase was collected, dried over Na_2SO_4, filtered and the solvent was evaporated under reduced pressure. Finally, the crude mixture was purified with column chromatography (1:1 EtOAc/cyclohexane) to obtain the purified product **2b** as yellowish oil (161 mg, 70% yield). ^{1}H NMR and ^{13}C NMR were identical as described above.

Biotransformation preparative scale (600 mL). Preparative scale biotransformation [prepared under inert atmosphere (N_2 5.0 bars, in a MBraun LABstar glove box)] was carried out on a 600 mL scale using a 2 L volume erlenmeyer flask as follows: freeze-dried MTase **I** (30 g, crude extract 50 mg/mL ≡ 1.95 mg/mL *dhaf*-MTase I) was rehydrated in *holo*-CP solution (300 mL, 500 µL/mL reconstituted *holo*-CP solution ≡ 21 mg/mL CP, obtained omitting the desalting step protocol). Donor *m*-**1b** (1.0 g, 10 mM) and acceptor **3c** (1.93 mL, 25 mM) dissolved in MOPS/KOH buffer (50 mM, pH 6.5, containing 150 mM KCl) were finally added accordingly. The reaction vessel was then sealed with a septum, covered with aluminum foil, and left shaking in an incubator at 30 °C and 120 rpm. After 24 h the quantitative conversion was observed by HPLC-UV. The crude mixture was then distributed in centrifuge vessels (100 mL per centrifuge vessel). The aqueous mixture was extracted with EtOAc (150 mL x7) by centrifugation (20 min, 4,000 rpm x3). The organic phase was collected, dried over Na_2SO_4, filtered and the solvent evaporated under reduced pressure. Finally, the crude mixture was purified by column chromatography (1:1 EtOAc/Cyclohexane) to obtain the purified product **2b** as yellowish oil (886.5 mg, 97% yield). ^{1}H NMR (300 MHz, MeOD) δ 6.72 – 6.62 (m, 2H), 6.52 (m, 21), 3.67 (t, J = 7.3 Hz, 2H), 2.66 (t, J = 7.3 Hz, 2H). ^{13}C NMR (75 MHz, MeOD) δ 146.1, 144.6, 131.7, 121.2, 117.0, 116.3, 64.6, 39.6.

Supplementary Results

Table S1. Thiol library screening. Product formation after 24 hours; average of three experiments.[a]

Acceptor	Demethylated product (2a) %	Substrate recovery (1a) %
**3a**	56.7±0.3	43.3±0.2
**3b**	>99±0.03	-
**3c**	>99±0.9	-
**3d**	20.9±0.2	79.0±0.5
**3e**	23.3±0.2	76.7±0.7
**3f**	2.4±0.02	97.6±0.5
**3g**	>99±0.7	-
**3h**	0.5±0.1	>99.5±0.3
**3i**	>99±0.6	-
**3j**	>99±0.7	-

[a] Donor: **1a** (10 mM); acceptor: **3a-j** (20 or 10 mM). Reaction conditions: 30 °C, 800 rpm, 50 mM MOPS buffer (KOH, 150 mM KCl, pH 6.5), MTase I (50 mg/mL CFE ≡ 1.95 mg/mL MTase I) and CP (500 µL/mL reconstituted holo-CP solution ≡ 21 mg/mL CP). Reaction volume 120 µL.

Table S2. Transformation with purified enzyme. Product formation after 24 hours; average of three experiments.[a]

Donor:Acceptor ratio	Demethylated product (2b) %	Isomerization product (*p*-1b) %	Substrate recovery (*m*-1b) %
1:5	83.6±0.04	6.6±0.6	9.7±0.7

[a] Donor: *m*-**1b** (10 mM); acceptor: **3b** (1=10 mM, 5=50mM). Reaction conditions: 30 °C, 800 rpm, 50 mM MOPS buffer (KOH, 150 mM KCl, pH 6.5), MTase I (13 mg/mL pure MTase I) and CP (400 µL/mL reconstituted pure 33 mg CP). Reaction volume 120 µL.

Table S3. Acceptor 3c concentration screening. Product formation after 24 hours; average of three experiments.[a]

Donor:Acceptor ratio	Demethylated product (2b) %	Isomerization product (*p*-1b) %	Substrate recovery (*m*-1b) %
1:5	>99±0.1	-	-
1:4	>99±0.6	-	-
1:3.5	>99±0.7	-	-
1:3	>99±0.2	-	-
1:2.5	>99±0.2	-	-
1:2	97.9±0.4	1.1±0.1	0.9±0.1
1:1.5	66.9±0.3	17.7±0.2	15.3 ±0.2
1:1	37.9±0.2	33.3±0.2	28.8±0.1

[a] Donor: *m*-**1b** (10 mM); acceptor: **3c** (different concentrations). Reaction conditions: 30 °C, 800 rpm, 50 mM MOPS buffer (KOH, 150 mM KCl, pH 6.5), MTase I (50 mg/mL CFE ≡ 1.95 mg/mL MTase I) and CP (500 µL/mL reconstituted holo-CP solution ≡ 21 mg/mL CP). Reaction volume 120 µL.

Table S4. Donor scope. Product formation after 24 hours; average of three experiments.[a]

Substrate *m*-1n	Demethylated product (2n) %	Isomerization product (*p*-1n) %	Substrate recovery (*m*-1n) %
m-**1b**	97.9±0.4	1.1±0.1	0.9±0.1
m-**1c**	93.9±0.6	-	6.1±0.4
m-**1d**	91.8±0.04	4.6±0.3	3.6±0.3
m-**1e**	70.5±0.7	9.8±0.3	19.7±0.5
m-**1f**	>99±0.2	-	-
m-**1g**	>99± 0.6	-	-
m-**1h**	97.1±0.2	2.9±0.1	-
m-**1i**	89.3±0.1	7.0±0.1	3.7±0.1

[a] Donor: *m*-**1b-i** (10 mM); acceptor: **3c** (20 mM). Reaction conditions: 30 °C, 800 rpm, 50 mM MOPS buffer (KOH, 150 mM KCl, pH 6.5), MTase I (50 mg/mL CFE ≡ 1.95 mg/mL MTase I) and CP (500 µL/mL reconstituted holo-CP solution ≡ 21 mg/mL CP). Reaction volume 120 µL.

Table S5. Donor scope. Product formation after 24 hours; average of three experiments.[a]

Substrate *p*-1n	Demethylated product (2n) %	Isomerization product (*m*-1n) %	Substrate recovery (*p*-1n) %
p-**1b**	96.2±0.4	-	3.7±0.02
p-**1c**	93.5±0.3	6.5±0.6	-
p-**1d**	91.8±0.04	3.6±0.3	4.6±0.3
p-**1e**	64.1±1.2	25.7±0.1	10.2±0.1
p-**1f**	98.8±0.8	0.6±0.1	0.6±0.1
p-**1g**	>99±0.1	-	-
p-**1h**	91.7±0.3	8.3±0.04	-
p-**1i**	87.9±0.3	4.2±0.05	7.9±0.1

[a] Donor: *p*-**1b-i** (10 mM); acceptor: **3c** (20 mM). Reaction conditions: 30 °C, 800 rpm, 50 mM MOPS buffer (KOH, 150 mM KCl, pH 6.5), MTase I (50 mg/mL CFE ≡ 1.95 mg/mL MTase I) and CP (500 µL/mL reconstituted holo-CP solution ≡ 21 mg/mL CP). Reaction volume 120 µL.

Table S6. Optimization of the protocol for scale up. Product formation after 24 hours; average of three experiments.[a]

mg CP (CFE)-Volume reconstitution solution	Incubation	Desalting	Conversion (1a) %
50 mg CP – 500 µL	2h	no	>99±1.7
50 mg CP – 400 µL	2h	no	>99±0.3
50 mg CP – 300 µL	2h	no	>99±1.0
50 mg CP – 500 µL	2h	yes	>99±0.6

[a] Donor: **1a** (10 mM); acceptor: **3c** (40 mM). Reaction conditions: 30 °C, 800 rpm, 50 mM MOPS buffer (KOH, 150 mM KCl, pH 6.5), MTase I (50 mg/mL CFE ≡ 1.95 mg/mL MTase I) and CP (500 µL/mL reconstituted holo-CP solution ≡ 21 mg/mL CP). Reaction volume 120 µL.

Calibrations Curves and Chromatograms

To quantify analytes by means of HPLC, calibration curves were established. Calibration standards (concentrations ranging from 1-50 mM) were prepared in MOPS/KOH buffer (50 mM, pH 6.5, 150 mM KCl) and treated in accordance with the biotransformation samples. Aliquots (10 µL) of samples were directly injected into HPLC and the area of the signal (absorbance unit mAu; y-axis) was plotted against the analyte concentration (mM, x-axis). Calibration curves for compounds: **1a**, **2a**, *m*- and *p*-**1d**, **2d**, *m*- and *p*-**1e**, **2e**, *m*- and *p*-**1f**, **2f**, *m*- and *p*-**1g**, **2g**, *m*- and *p*-**1h** and **2h** were taken from literature.[1] The calibrations for the rest of the substrate scope is here reported.

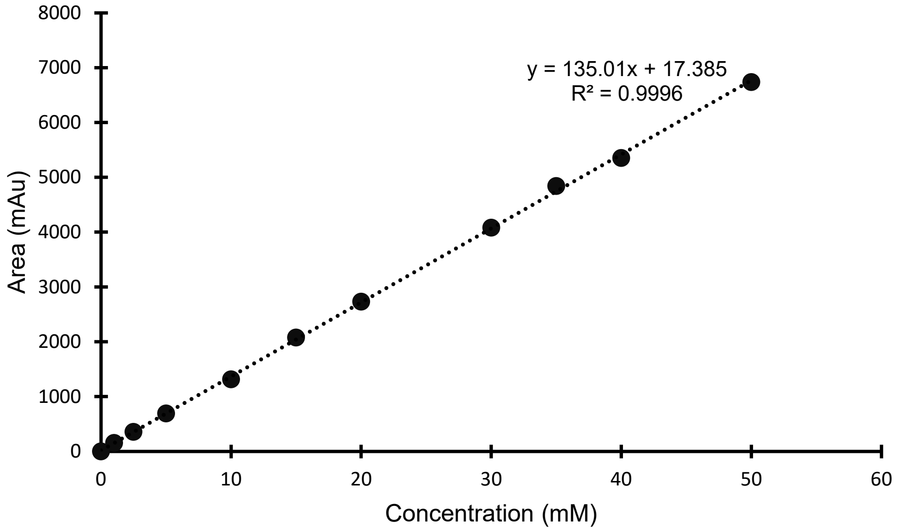

Figure S3. Calibration curve for 5-(2-hydroxyethyl)-2-methoxyphenol (*p*-**1b**).

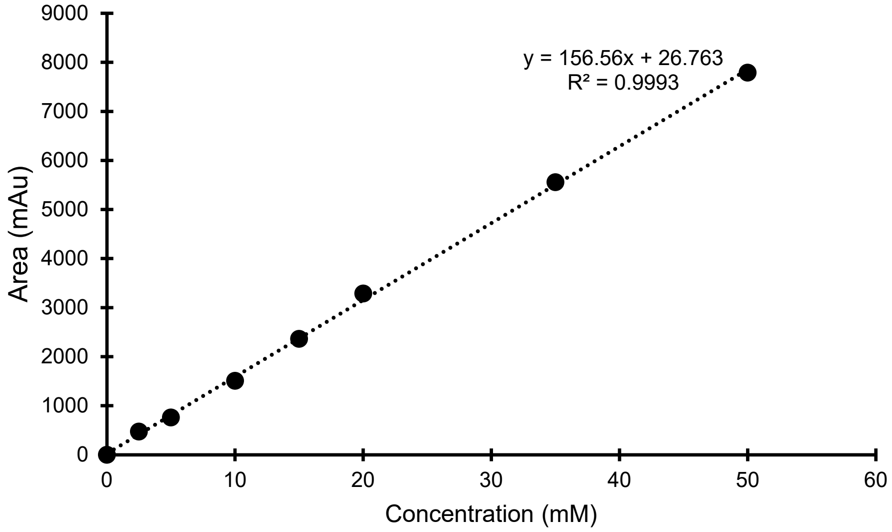

Figure S4. Calibration curve for hydroxytyrosol (**2b**).

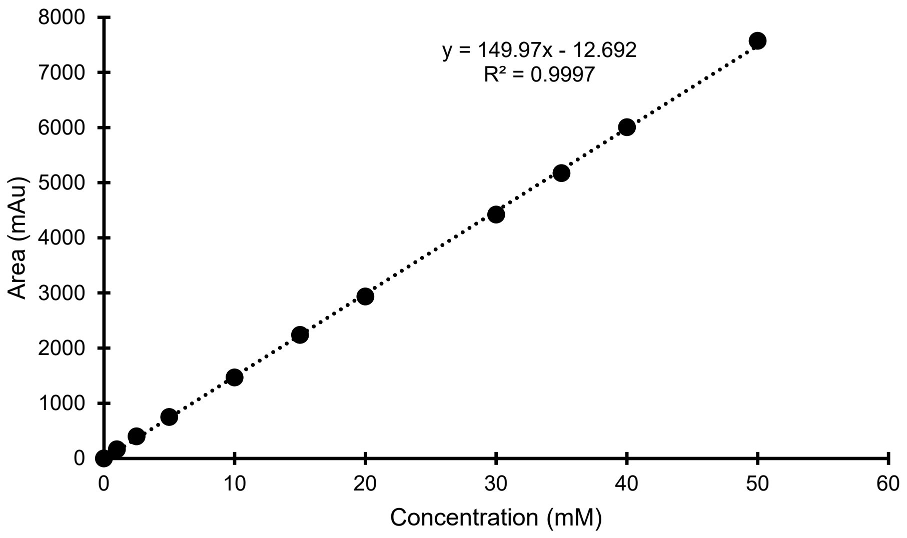

Figure S5. Calibration curve for homovanillyl alcohol (*m*-**1b**).

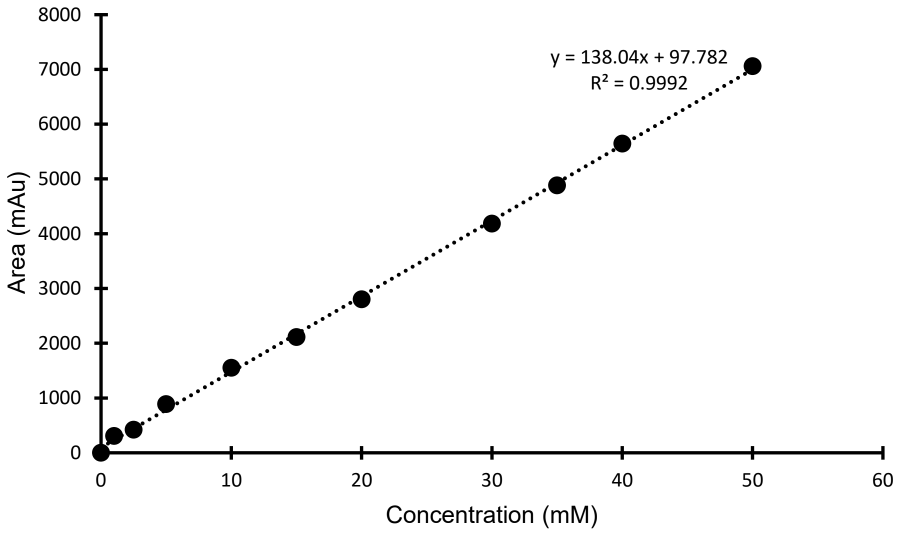

Figure S6. Calibration curve for 2-methylcatechol (**2i**).

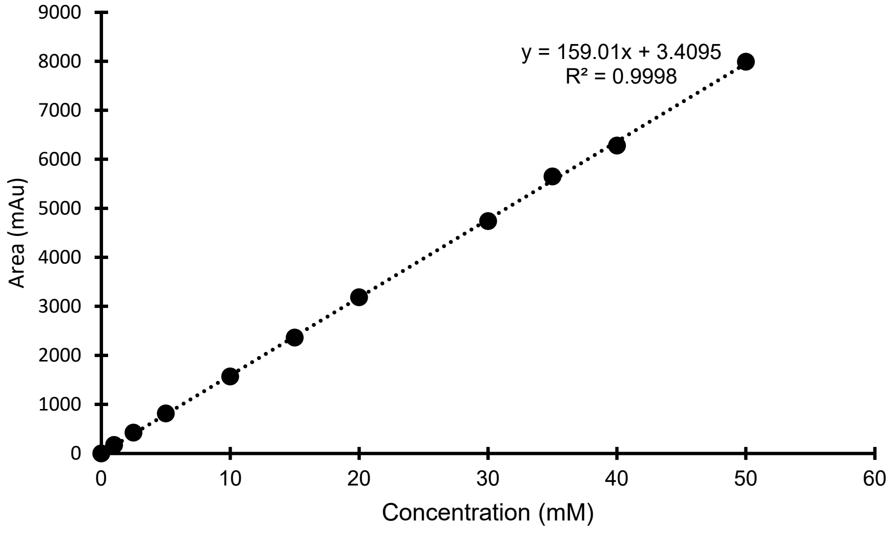

Figure S7. Calibration curve for 2-methoxy-4-methylphenol (*m*-**1i**).

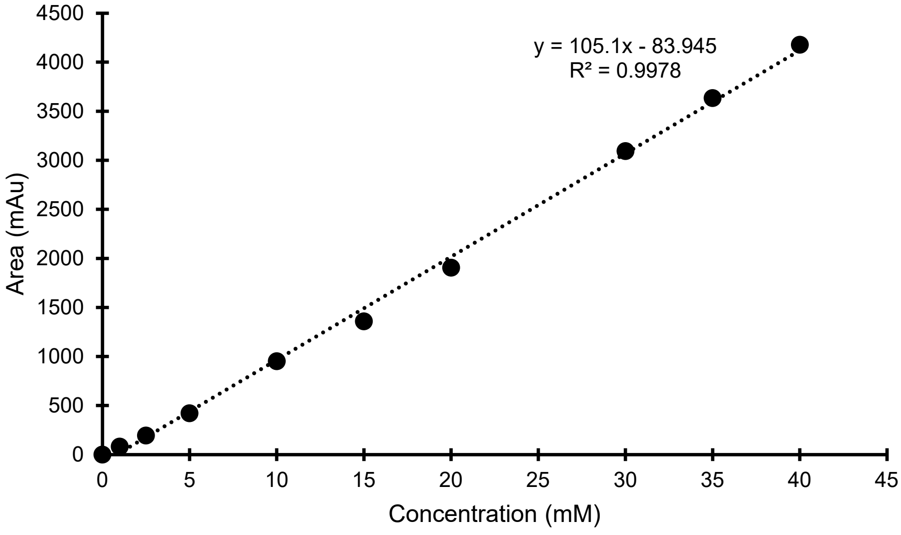

Figure S8. Calibration curve for 2-methoxy-5-methylphenol (*p*-**1i**).

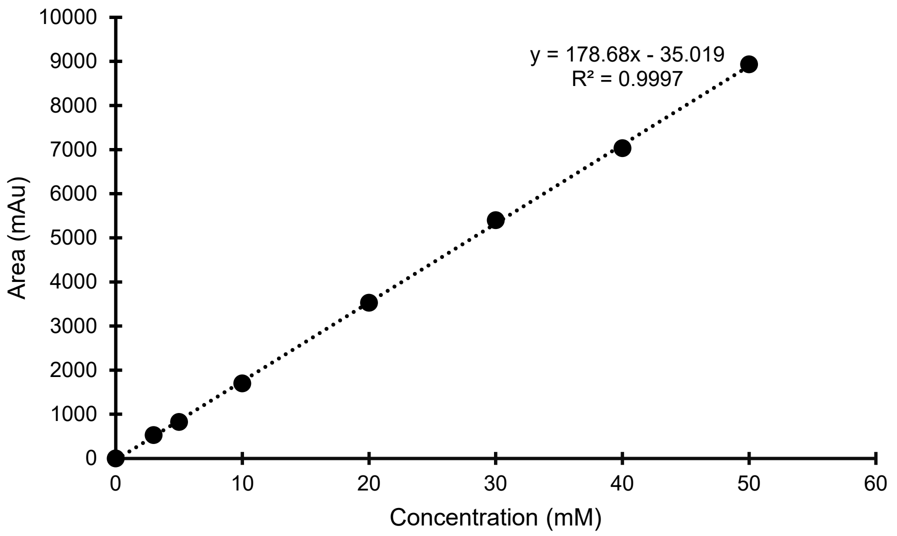

Figure S9. Calibration curve for 4-ethyl-2-methoxyphenol (*m*-**1c**).

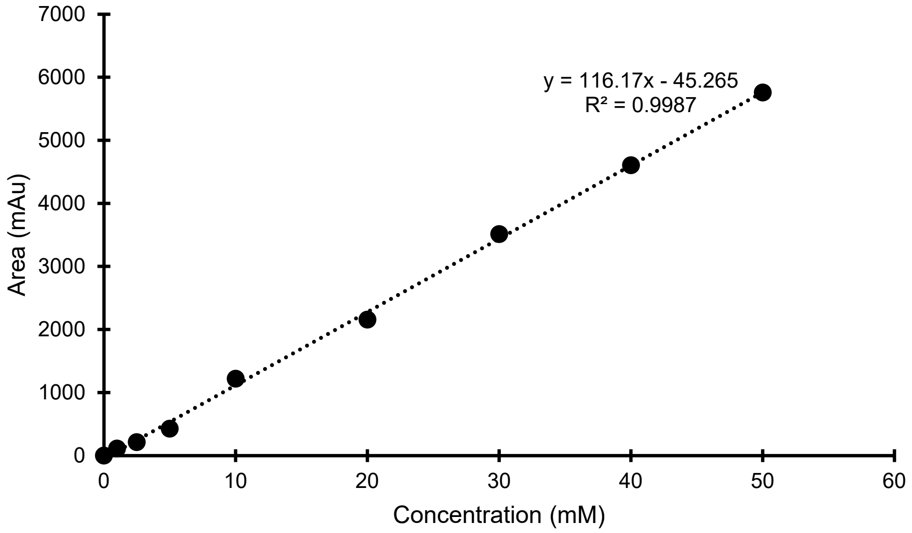

Figure S10. Calibration curve for 5-ethyl-2-methoxyphenol (*p*-**1c**).

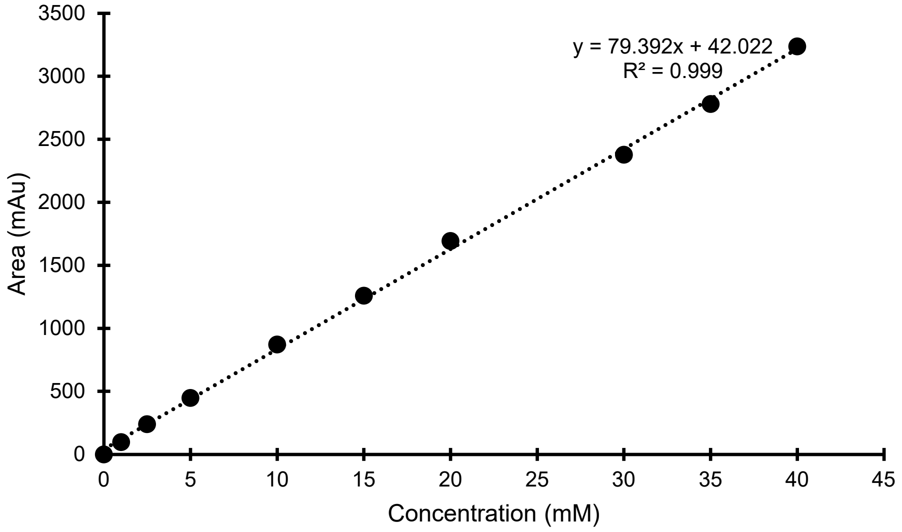

Figure S11. Calibration curve for 4-ethylcatechol (**2c**).

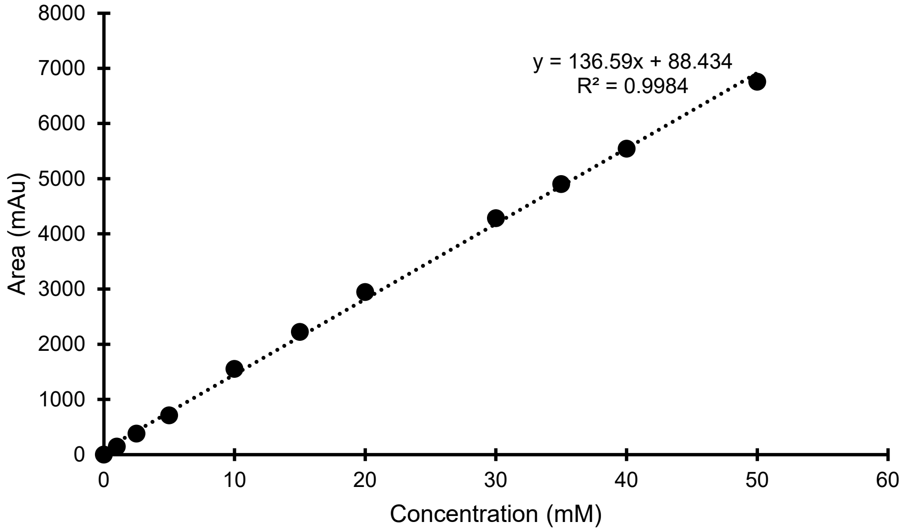

Figure S12. Calibration curve for 4-hydroxy-3-methoxyacetophenone (*m*-**1d**).

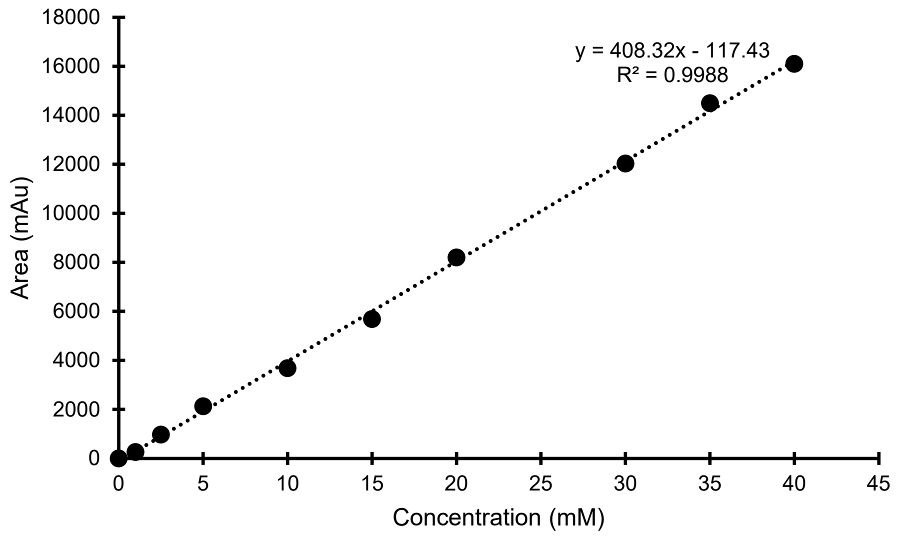

Figure S13. Calibration curve for 4-hydroxy-3-methoxyacetophenone (*p*-**1d**).

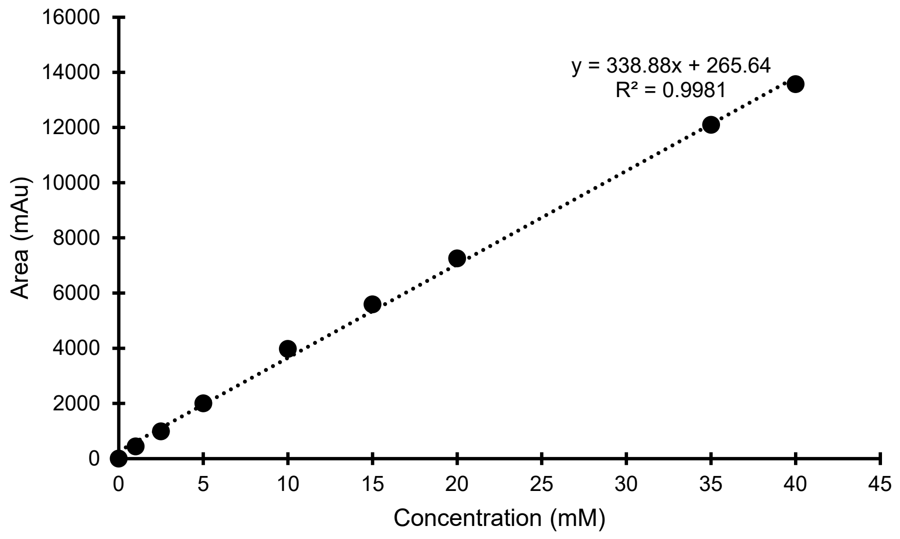

Figure S14. Calibration curve for 3,4-dihydroxyacetophenone (**2d**).

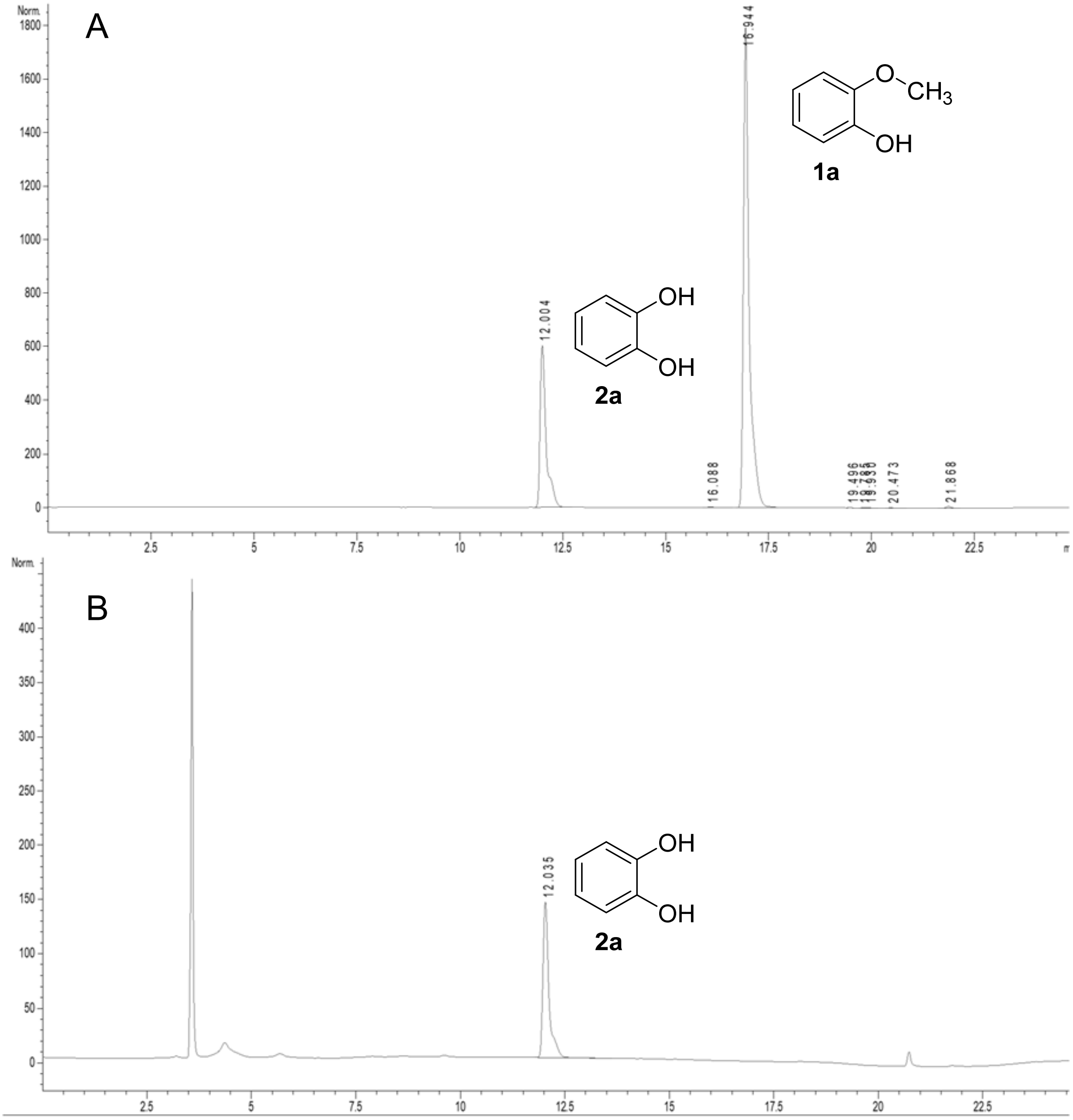

Figure S15. Example of HPLC chromatograms for the demethylation reaction to afford **2a**. (**A**) Commercially available references (**2a** rt 12.0, **1a** rt 16.9). (**B**) The demethylation reaction of guaiacol **1a** (10 mM) with the acceptor **3c** (50 mM) leading to the quantitative conversion towards catechol **2a**.

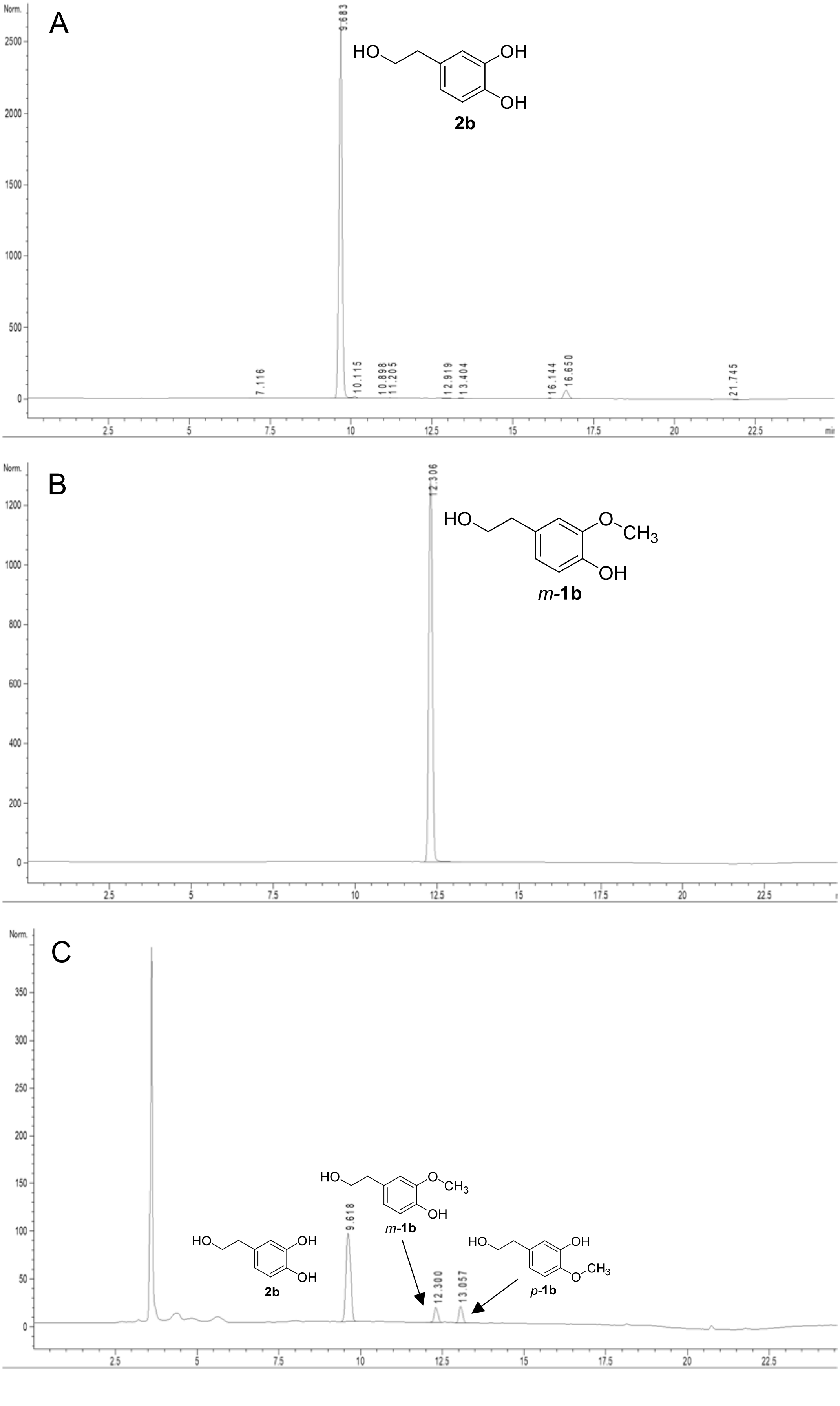
A
Norm.
HO
OH
OH
2b
9.683
7.116
10.115
10.898
11.205
12.919
13.404
16.144
16.650
21.745
B
Norm.
HO
O
CH3
OH
m-1b
12.306
C
Norm.
HO
OH
OH
2b
9.618
HO
O
CH3
OH
m-1b
12.300
13.057
HO
OH
O
CH3
p-1b

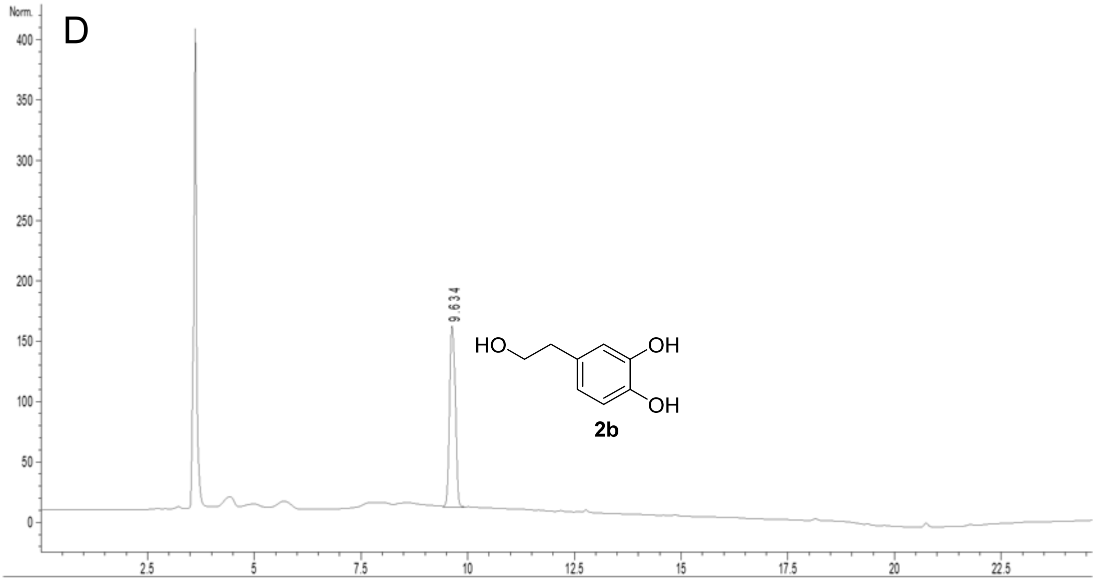

Figure S16. Example of HPLC chromatograms for the demethylation reaction to afford **2b**. (**A-B**) Commercially available references (**2b** rt 9.7, *m*-**1b** rt 12.3). (**C**) The demethylation reaction of homovanillyl alcohol *m*-**1b** (10 mM) with acceptor **3c** (15 mM) leading to a mixture of isomers (*m*-**1b**, *p*-**1b**) and hydroxytyrosol (**2b**). (**D**) The demethylation reaction of homovanillyl alcohol alcohol *m*-**1b** (10 mM) with acceptor **3c** (50 mM) leading to quantitative conversion towards the desired product hydroxytyrosol (**2b**).

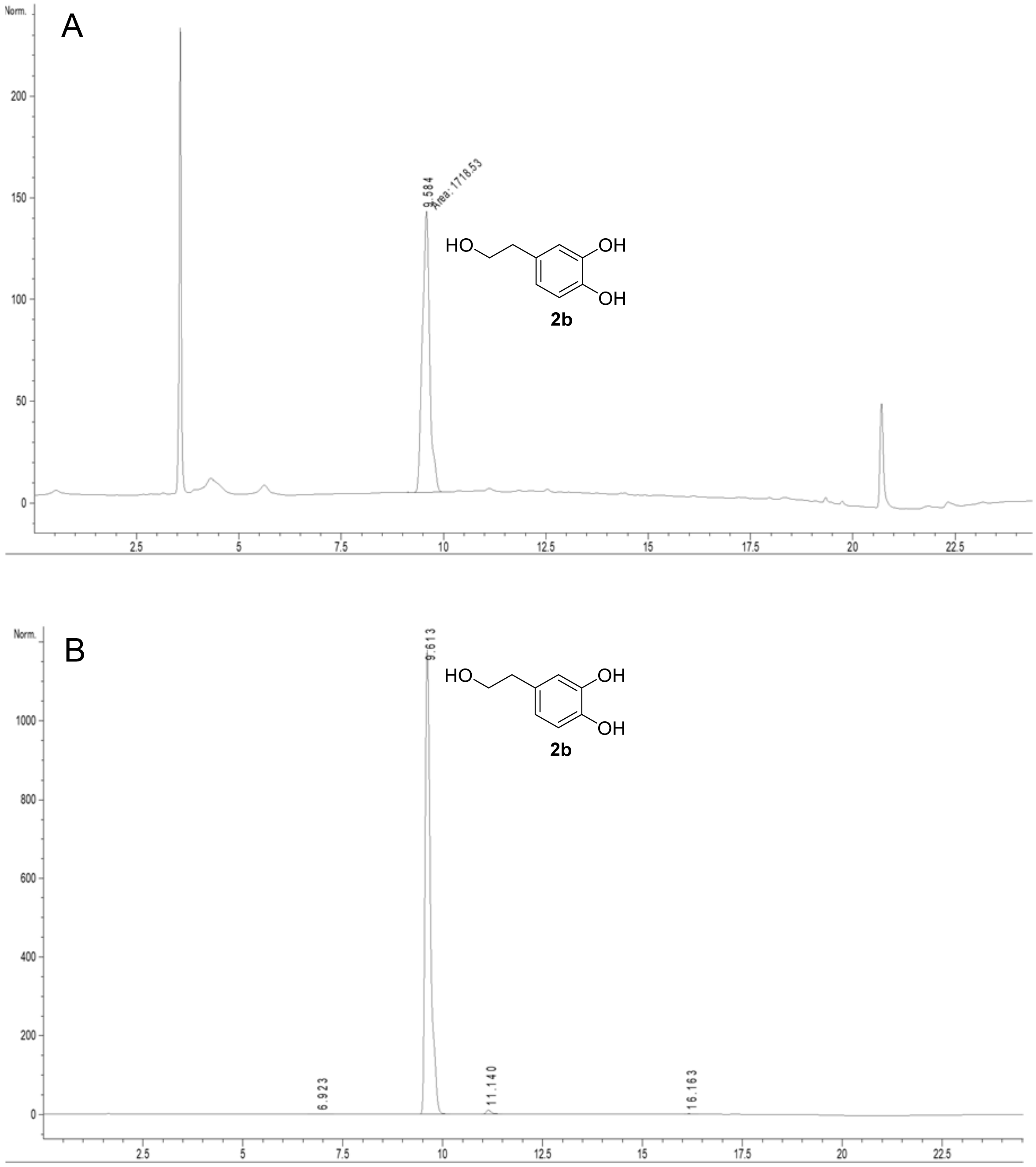

Figure S17. Example of HPLC chromatograms for the demethylation reaction to afford **2b**. (**A**) The scale up demethylation reaction (600 mL) of homovanillyl alcohol *m*-**1b** (10 mM) with acceptor **3c** (25 mM) leading to quantitative conversion towards Hydroxytyrosol (**2b**). (**B**) Hydroxytyrosol **2b** after the isolation and purification.

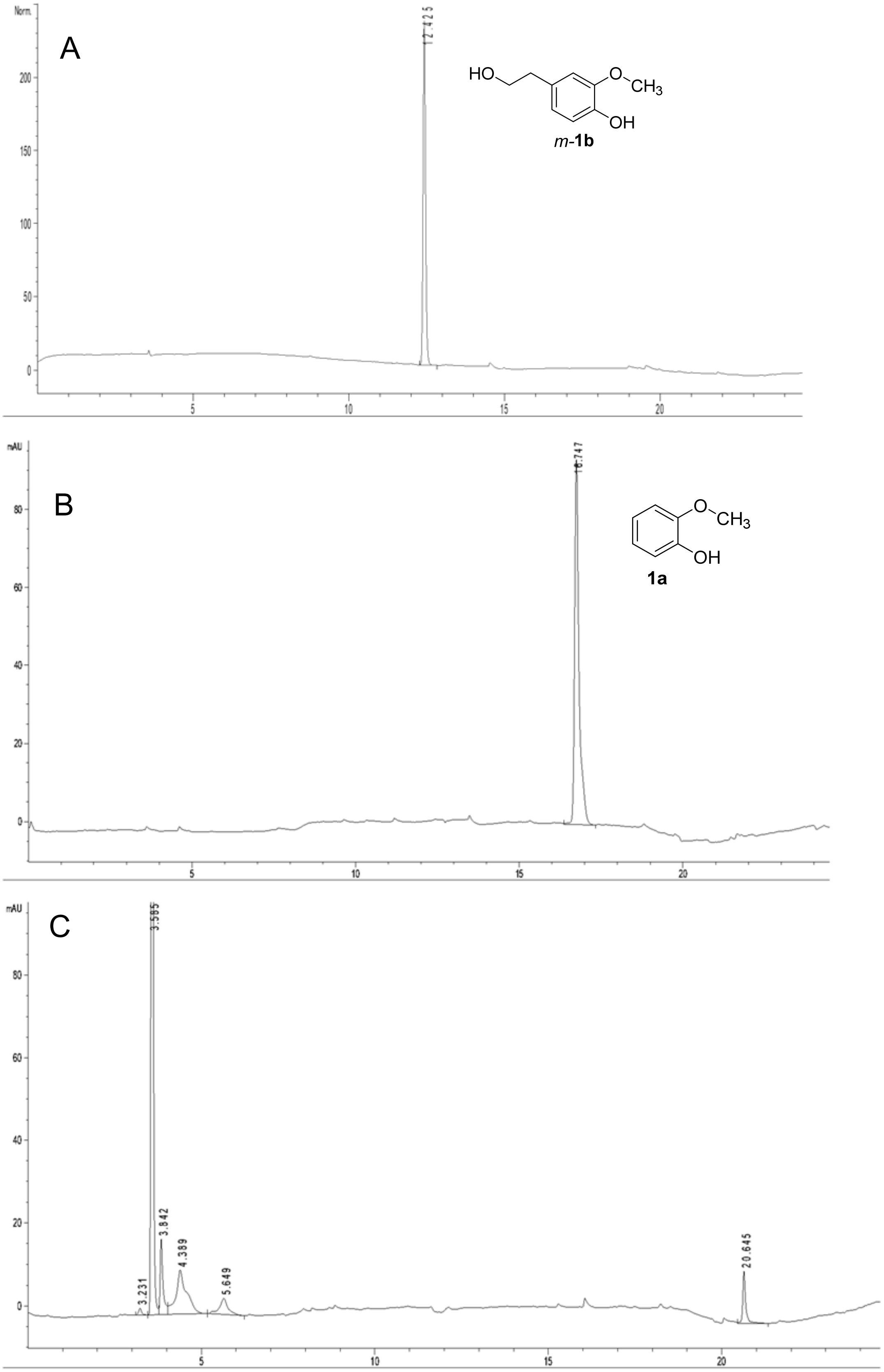

Figure S18. Blank reactions in the absence of catalyst. (**A**) Only the peak for homovanillyl alcohol *m*-**1b** was detected in the blank reaction in which *m*-**1b** (10 mM) and acceptor **3c** (20 mM) at pH 9 were left stirring at 30 °C, 800 rpm for 24 h in the absence of catalysts. (**B**) Only the peak for guaiacol **1a** was detected in the blank reaction in which **1a** (10 mM) and acceptor **3c** (20 mM) at pH 6.5 were left stirring at 30 °C, 800 rpm for 24 h in the absence of catalysts; (**C**) blank reaction in the absence of donor and acceptor; the peaks in the chromatogram can be ascribed to compounds present in the CFE.

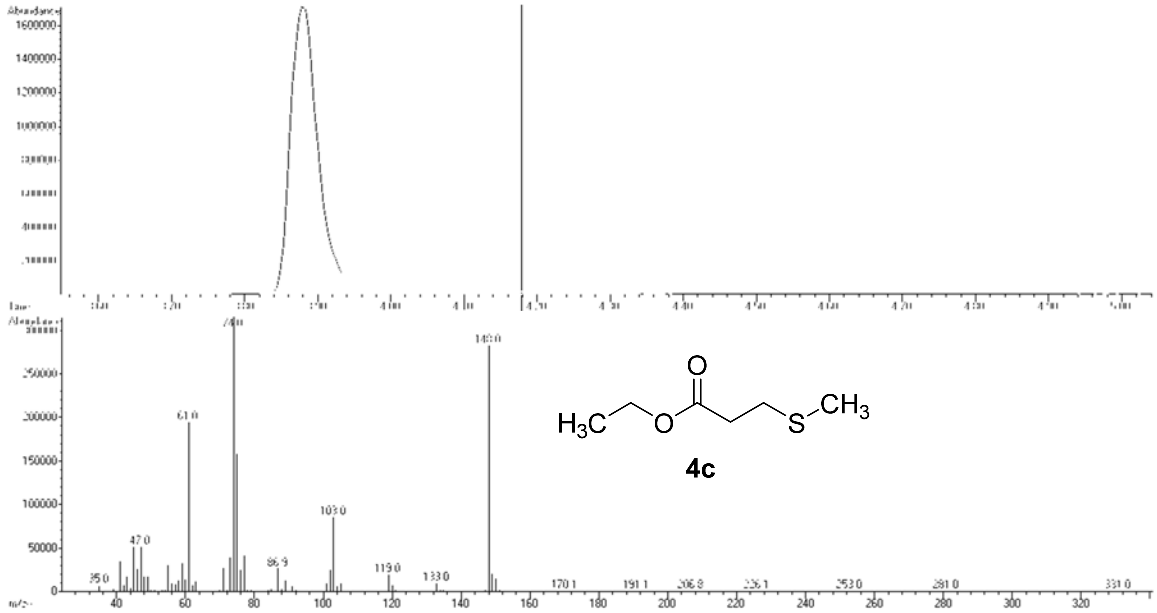

4c

Figure S19. Example of GC-MS spectra for the demethylation reaction. The peak for the methylated acceptor **4c**.

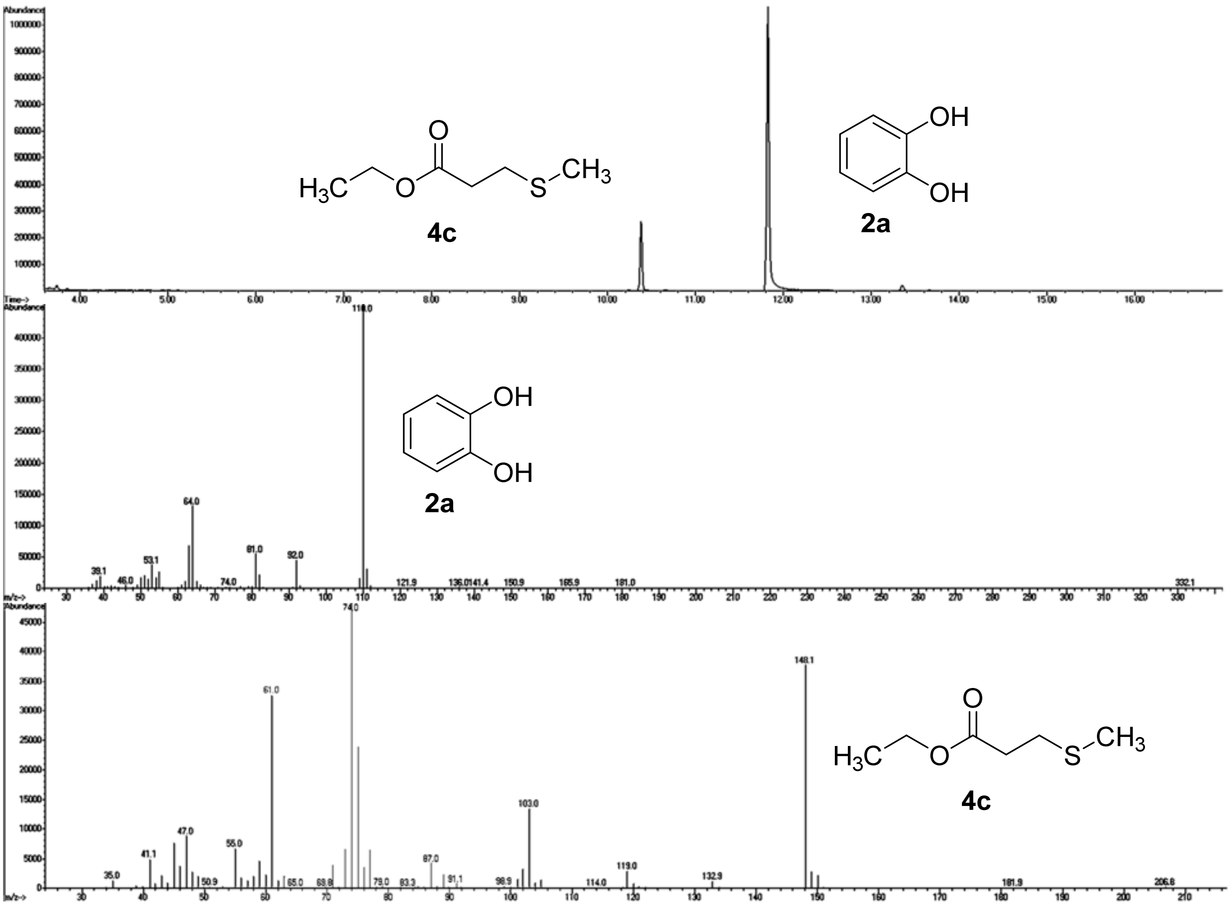

Figure S20. GC-MS spectra testing the hydrolysis of **4c** and reversibility of the reaction employing **4c** as methyl donor and catechol **2a** as methyl acceptor. Only the peaks for the methylated acceptor **4c** and catechol **2a** were observed.

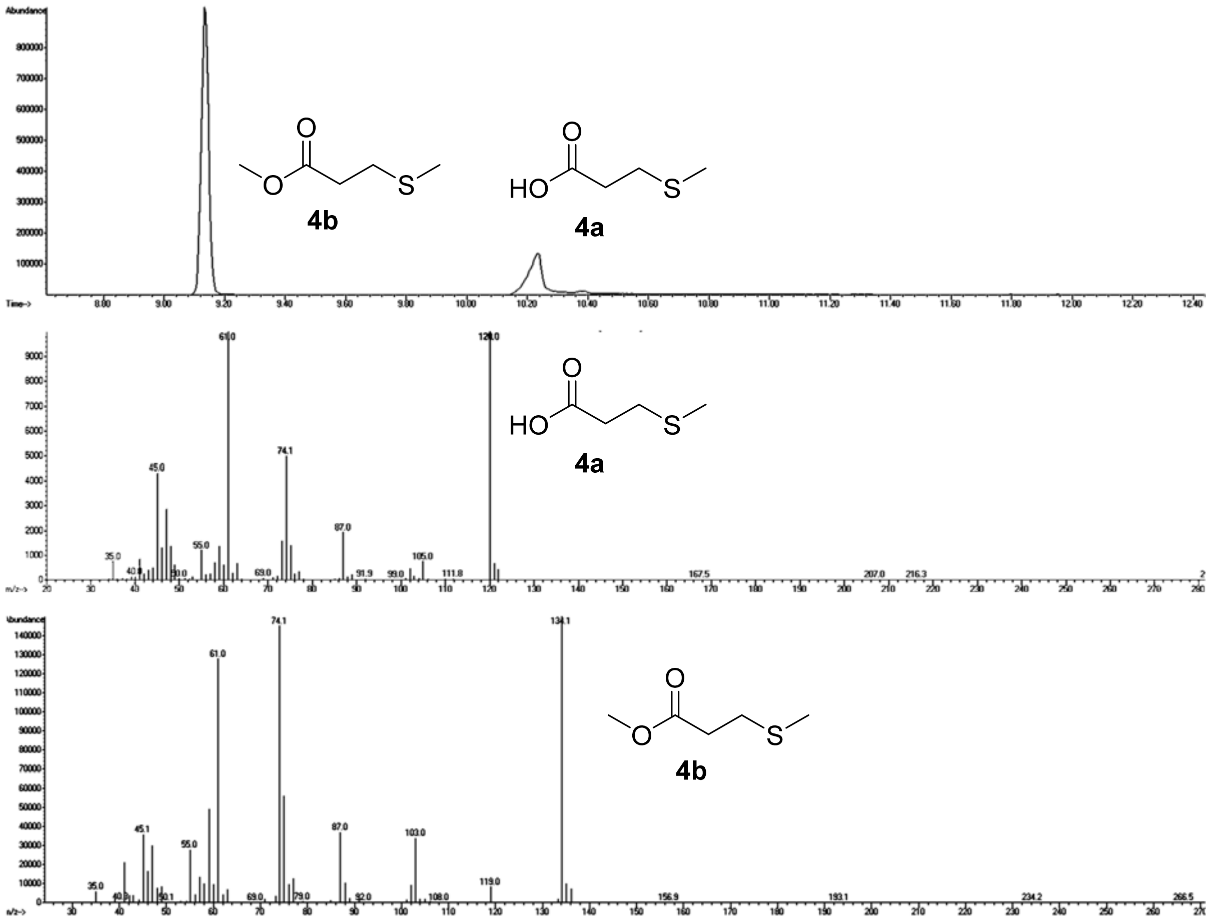

Figure S21. GC-MS spectra testing the hydrolysis of **4b** promoted by the CFE. Both the peaks for the methylated acceptor **4b** and the product of hydrolysis **4a** were observed.

NMR

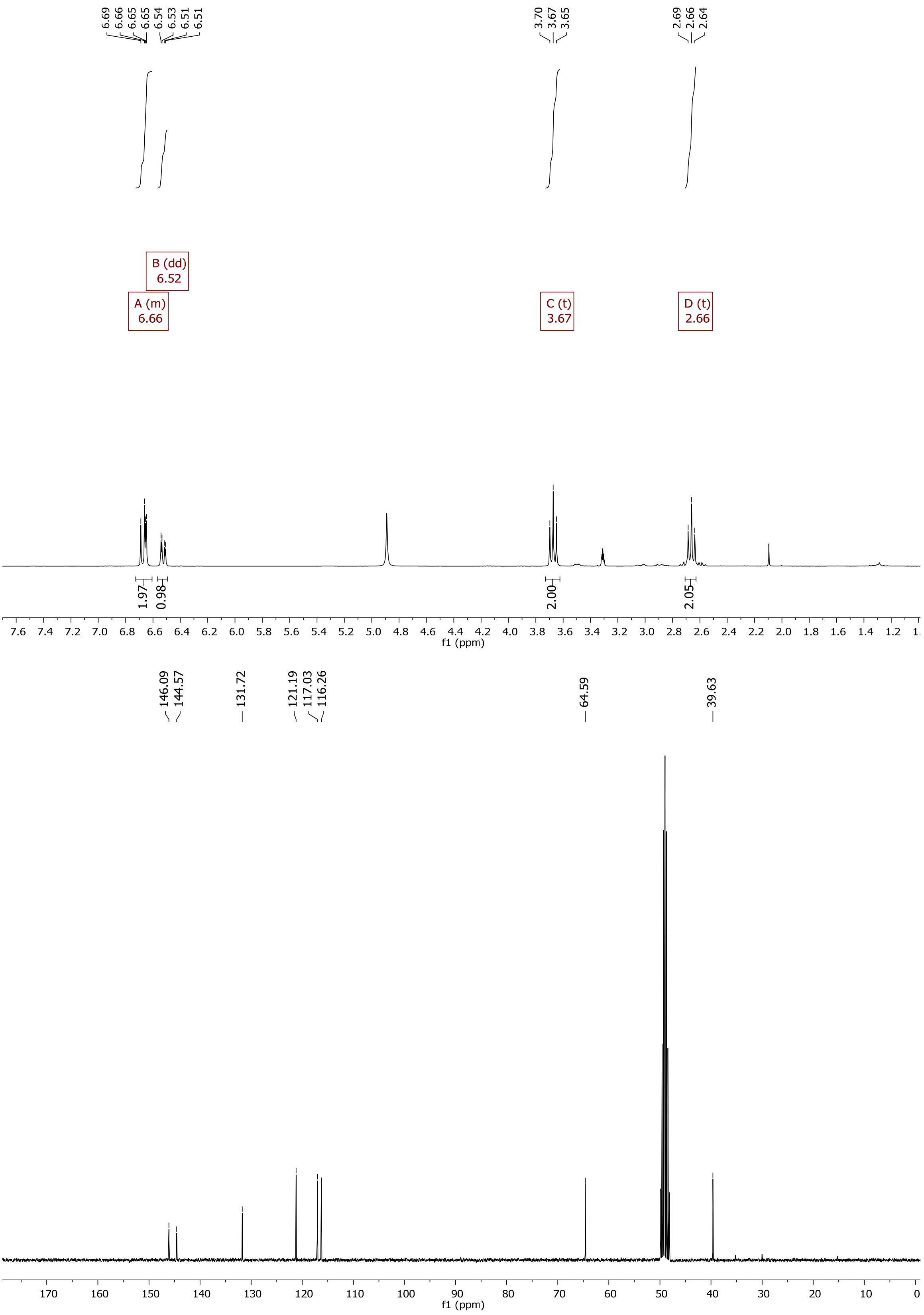

Figure E18. ^{1}H NMR and ^{13}C NMR hydroxytyrosol **2b** from preparative scale (600 mL). ^{1}H NMR (300 MHz, MeOD) δ 6.72 – 6.62 (m, 2H), 6.52 (dd, *J* = 8.0, 2.1 Hz, 1H), 3.67 (t, *J* = 7.3 Hz, 2H), 2.66 (t, *J* = 7.3 Hz, 2H). ^{13}C NMR (75 MHz, MeOD) δ 146.0, 144.5, 131.7, 121.1, 117.0, 116.2, 64.5, 39.6.

References

[1] J. E. Farnberger, N. Richter, K. Hiebler, S. Bierbaumer, M. Pickl, W. Skibar, F. Zepeck, W. Kroutil, *Commun. Chem.* **2018**, *1*, 82.

[2] C. Grimm, M. Lazzarotto, S. Pompei, J. Schichler, N. Richter, J. E. Farnberger, M. Fuchs, W. Kroutil, *ACS Catal.* **2020**, *10*, 10375-10380.
